# Temporal Dashboard Gaze Variance (TDGV) Changes for Measuring Cognitive Distraction While Driving

**DOI:** 10.3390/s22239556

**Published:** 2022-12-06

**Authors:** Cyril Marx, Elem Güzel Kalayci, Peter Moertl

**Affiliations:** Virtual Vehicle Research GmbH, 8010 Graz, Austria

**Keywords:** driver monitoring, cognitive distraction, gaze variance, eye tracking, behavioral regularity

## Abstract

A difficult challenge for today’s driver monitoring systems is the detection of cognitive distraction. The present research presents the development of a theory-driven approach for cognitive distraction detection during manual driving based on temporal control theories. It is based solely on changes in the temporal variance of driving-relevant gaze behavior, such as gazes onto the dashboard (TDGV). Validation of the detection method happened in a field and in a simulator study by letting participants drive, alternating with and without a secondary task inducing external cognitive distraction (auditory continuous performance task). The general accuracy of the distraction detection method varies between 68% and 81% based on the quality of an individual prerecorded baseline measurement. As a theory-driven system, it represents not only a step towards a sophisticated cognitive distraction detection method, but also explains that changes in temporal dashboard gaze variance (TDGV) are a useful behavioral indicator for detecting cognitive distraction.

## 1. Introduction

Driver distraction is one of the most common factors reported when it comes to crashes or safety critical events. Previous research has shown that 5% to 25% of accidents can be attributed to the involvement of driver distraction [1]. Typically, visual distraction (when the driver’s gaze is off the road and oriented towards driving-irrelevant visual information) and cognitive distraction (when the driver’s mind is off the road and their thoughts are oriented towards driving-irrelevant information) are distinguished [1,2]. It has been shown that when drivers are engaged in complex visual and/or manual secondary tasks, they have a near-crash/crash risk three-times higher than attentive drivers [3].

Different definitions of driver distraction exist. Engström et al. [4] proposed two different type of inattention: (a) insufficient attention and (b) misdirected attention. They defined driver distraction as situations in which the driver’s resources are allocated “to a non-safety critical activity while the resources allocated to activities critical for safe driving do not match the demands of these activities” (p. 35). This means that distraction occurs in situations where attention is taken away from driving-related activities and towards non-safety-related activities. Similarly, Regan et al. [5] proposed a taxonomy where distraction is “the diversion of attention away from activities critical for safe driving toward a competing activity, which may result in insufficient or no attention to activities critical for safe driving” (p. 1776) [6].

In terms of cognitive distraction, those definitions resulted in two different concepts, namely mind-wandering and external distraction. The former can be defined as a form of cognitive distraction where “an individual’s attention is shifted from external events to internal thoughts and is thus decoupled from perceptual inputs” [7]. In contrast, external distraction means that attention “has shifted away from goal relevant information due to external non goal relevant stimuli…” [7], as in talking to another person while driving. Both differ in the circumstances in which they appear. While mind-wandering is mainly present in situations of low perceptual complexity and little external sensory input, external cognitive distraction is a result of highly complex situations where many different sources give input to the driver [8].

### 1.1. Measuring Driver’s Distraction

There are several driver distraction detection algorithms that utilize driving performance measurements, as well as physiological methods, especially gaze behavior, to categorize distracted driving (see e.g., [1,9,10] for overviews).

A very simple assumption is that a driver is (visually) distracted once he or she is not looking on the street (or driving-related areas, such as the mirrors or the speedometer) for some time. Previous findings showed that the time during which the focus is off the road is typically at a maximum of approximately 1.6 s ([11] cited after [12]), and that glances off the road longer than 2 s increase the risk of crashes significantly [3]. Many algorithms, therefore, use a “gaze-off-the-road” criteria to detect distraction (e.g., [3,5]; see also [10]). For instance, the AttenD algorithm utilizes an internal timer of two seconds that is emptied when looking away from the road and filled back up when the gaze is laid on the road again. This ensures road gazes that are, at minimum, long enough for the driver to process the surrounding information [13].

For cognitive distraction detection, gaze parameters such as the mean fixation durations (intervals in which eyes are focused on one location) and the length, as well as the speed of saccades (rapid eye movements between fixations), have been found to be impactful, although the direction of change—longer or shorter for fixation durations and saccade lengths—is often less clear [2,3,4]. Finally, gaze has also been shown to be more concentrated for a gaze under high, as compared to low, cognitive load [4,14]; especially, horizontal gaze movement is reduced under high cognitive load [15]. Other models incorporate multiple physiological parameters besides eye tracking information, such as heart rate [16].

Those approaches are often difficult to measure because they require either multiple parameter input from different sources, or they are not viable to implement in a moving vehicle. The latter, for example, reduces the accuracy of fixation detection due to the constant vibration of the eye tracking device. The present paper introduces the change of temporal dashboard gaze variance (TDGV) as a distraction detection method addressing those issues due to the ease of measuring it in a real moving vehicle.

### 1.2. Measuring Cognitive Driver Distraction through Temporal Dashboard Gaze Variance (TDGV) Changes

#### 1.2.1. Theory behind Temporal Dashboard Gaze Variance

Previous neuroscientific and psychological work found an inverse connection between temporal regularity in behavior and cognitive load [17,18,19]. Increased load from distracting stimuli leads to a reduction of temporal regularity and time-synchronized behavior while performing a task, such as playing musical notes for certain intervals of time, even for experts in their field [17]. Although well researched in neuroscience, the ability to detect cognitive driver distraction with this reduction of the regularity of certain behavior, such as safety-critical gaze shifting behavior, has not yet been reported.

The regular validation of safety-critical information from the environment, such as one’s current speed, is necessary for driving. Keeping regularity in such behavior requires the temporal control of the driver’s gaze. A reduction in the temporal control of this behavior under cognitive distraction leads to the hypothesis that cognitive distraction can be detected by measuring the change in the regularity of dashboard gazes, or, in other words, changes in the temporal dashboard gaze variance (TDGV).

#### 1.2.2. Calculation of Distraction Detection with TDGV

The method approaches the issue of detecting cognitive distraction by measuring the regularity of gaze frequency at the dashboard, since checking the speed is a safety-critical behavior that a driver performs regularly. If this regularity suddenly starts to decrease and the variance in the behavior suddenly increases above a certain threshold, the method detects a cognitive distraction.

At the start of measuring, the method is initialized by creating an empty list of size n, where n represents the number of past dashboard gazes to include in the standard deviation calculations. To obtain information about the gaze variance, the detection method performs the following specific set of operations for every measuring time point t.

gaze_list(t)

If the driver looks at the dashboard, the current time is stored. If they do not look at the dashboard, the difference between the current time and the stored time of the last dashboard gaze is calculated. This value is stored in the initially created list and updated for every new datapoint until the driver looks at the dashboard again. After this happens, the value is ultimately stored and will not be overwritten again. If the driver looks away from the dashboard now, a new value of the same kind is stored in the list and updated until they look at the dashboard again. This list grows with every new dashboard gaze until it reaches a size of n, after when the oldest value is removed from the list and exchanged with the newest value with each new gaze at the dashboard. The listed algorithm is seen below in Algorithm 1.
**Algorithm 1**x ← −1 // Current position in gaze_list n ← 10 // Size of gaze_list **REPEAT:** **IF** current gaze is on dashboard:  **IF** previous gaze is not on dashboard:   x ← x + 1   **IF** x ≥ n:    x ← 0   **END IF**  **END IF**  gaze_time ← current time  **ELSE:**  **IF** x ≥ 0:   gaze_list[x] ← current time–gaze_time  **END IF** **END IF**  current_TDGV ← standard deviation of gaze list continue to next gaze datapoint **UNTIL** end of measurement

For every datapoint directly after the list is updated, the standard deviation of all values inside the list is calculated to calculate the current TDGV value.

σ(t)=σ(list(t))

Now the average over the last 2 s of this standard deviations is calculated. This step is necessary to smooth out irregularities caused by abrupt changes in the calculated values. Tests with the algorithm in the following two studies show that leaving out this step can lead to less reliable and interrupted detection due to noise.

m=2∗frequency of data in Hz

$$\sigma_{\bar{x}}\left(t\right)=\frac{1}{m}\overset{m-1}{\underset{i=0}{\sum }}\;\sigma \left(t-i\right)$$

The difference between the current averaged value and the last one is calculated to obtain the situational change of the standard deviation of dashboard gazes. This results in a derivative approximation of the standard deviation data.

$$\Delta \sigma \left(t\right)=\sigma_{\bar{x}}\left(t\right)-\sigma_{\bar{x}}\left(t-1\right)$$

If the resulting value is greater than or equal to 0, it accumulates over time until the current value of the derivation is negative. If this happens, the accumulated value stays 0. This approach allows for detecting only increases of standard deviation and ignoring reductions.

Δσ(t)≥0→ Δσ(t)cum=Δσ(t)+Δσ(t−1)

Δσ(t)<0→ Δσ(t)cum=0

If this accumulated value exceeds a certain threshold (T), the method detects cognitive distraction. In the following studies, the threshold was obtained by calculating the maximum TDGV change of an initial baseline drive of a driver.

Δσ(t)cum>T → DISTRACTION

The current paper investigates the described TDGV change as a method to detect the cognitive distraction of drivers. Two studies were performed. The first study is designed to investigate if these changes in TDGV on the dashboard/speedometer exist while driving, and if the proposed method can be used reliably for cognitive distraction detection. Furthermore, we investigate if such a method explicitly detects cognitive distraction, or if it is also triggered by visual distraction. The second study builds onto the results of the first study to investigate how accuracy can be increased and how individual differences influence the method’s performance.

## 2. Study 1: Investigation of Temporal Regularity Reduction and Developing the Distraction Detection

### 2.1. Materials and Methods

#### 2.1.1. Design

We conducted the first study with seven participants to explore the proposed temporal dashboard gaze variance (TDGV) change metric for cognitive distraction detection. The participants were driving in a passenger car on a motorway while performing several secondary tasks and while their gaze behavior was observed. The primary task was to safely drive the vehicle. During the whole drive, participants were not allowed to activate driving assistance systems, such as cruise control or lane keeping assistance.

**Figure 1:**
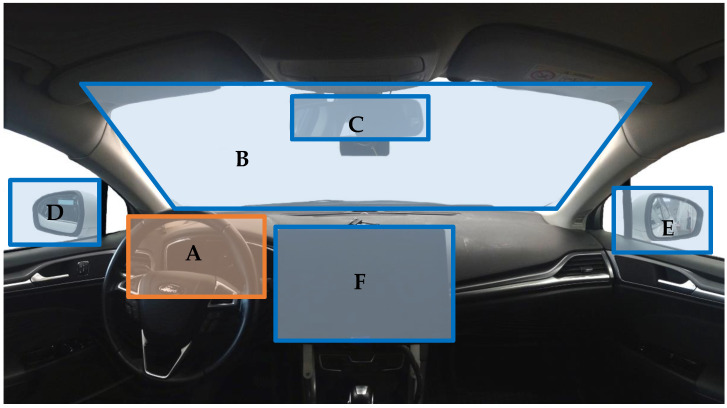
Areas of interest (AOI) in the experimental setup. A: dashboard; B: windshield; C: rear mirror; D: left mirror; E: right mirror; F: SURT Tablet. Only the dashboard AOI is used in analysis. The others were used for better evaluation of data validity.

To monitor the gaze of the drivers, we used a SmartEye^®^ eye tracking system consisting of two infrared cameras. This gaze data can be compared to the position of a predefined set of areas of interest (see Figure 1), such as the dashboard, to obtain an estimation of what driving-relevant areas the subject looks at during any given moment. Inside the vehicle were three other people monitoring the data collection and conducting the study. In the passenger seat was a trained test driver to intervene in case of a critical safety situation.

#### 2.1.2. Secondary Tasks

**Figure 2:**
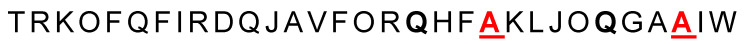
Example for Q3A-MEM, where the participant has to react to an A each time it succeeds a Q with two other random letters in between. Participants would have to react to each highlighted A in this example case.

To induce cognitive distraction in the participants, we used a variant of the Auditory Continuous Performance Task [20] (ACPT), which consists of working memory tasks with adjustable difficulty. We specifically used the Q3A-MEM variant. This task requires the subject to listen to a special cue inside several spoken letters and respond by saying “now” aloud when a certain target letter occurs three letters after the cue. To accomplish this task, participants had to actively keep a sequence of letters in memory, which induces cognitive load. Figure 2 shows an example of how the task works.

**Figure 3:**
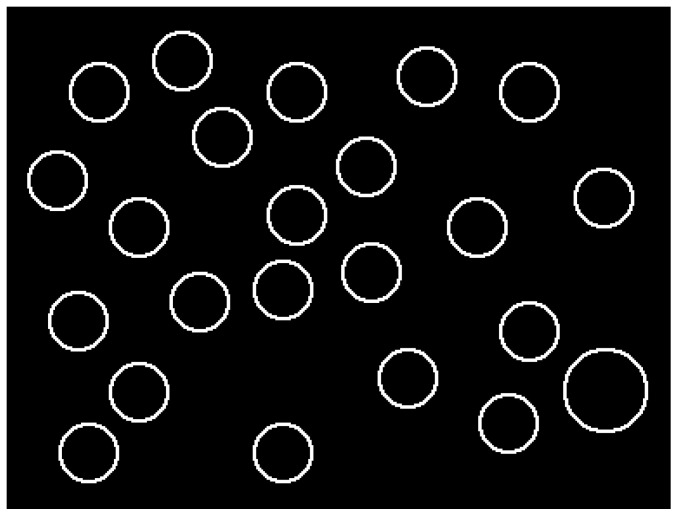
Schematic example task of the SuRT. Participants had to react to the larger circle.

To obtain an understanding of whether TDGV changes solely detect cognitive distraction, or if it also is influenced by visual distraction, we induced visual distraction as a separate condition by asking participants to perform the Surrogate Reference Task [19] (SuRT). Participants had to monitor a set of circles on the mounted touch screen, identify a slightly larger circle, and touch it with their finger. Directly afterwards, a new set of similar cues with a slightly changed stimulus in between was presented (see Figure 3).

#### 2.1.3. Study Procedure

**Figure 4:**
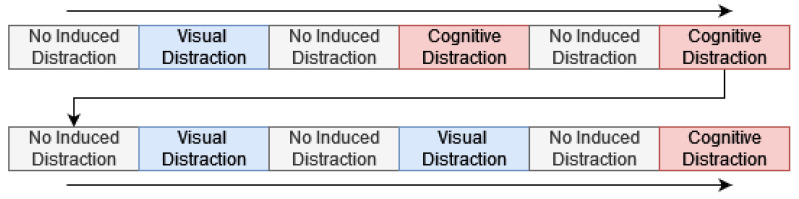
Experimental segments during study 1. Each segment took approximately one minute.

After giving their written informed consent for participating in the study, participants were instructed to drive a specific route and return to the starting point afterwards, which took about 40 min in total per trial. After they were guided to the highway, they drove for a couple of minute freely before the data collection and the study began. With a constant speed of about 100 km/h, the participants conducted various side tasks while driving. Each side task took about one minute and was given three times in total with a segment of undistracted driving in between. Figure 4 depicts the order of the experimental conditions.

#### 2.1.4. Analysis

As a first step, we investigated the assumption underlying our hypothesis that temporal variance in speedometer gazes increases under cognitive distraction. This was done by calculating the average standard deviation for all participants grouped by the experimental condition and comparing the values against each other.

To analyze the hypothesis that TDGV changes are able to detect cognitive distraction in drivers, we calculated the TDGV distraction metric for every driver during the whole drive. We then compared how many segments with and without secondary tasks were marked as distracted at least once. With this information, we calculated the accuracy as the percentage of correctly detected cases relative to all cases. However, this parameter alone does not provide descriptive information about the strengths and weaknesses of the distraction detection method. For this, we calculated the sensitivity, representing the percentage of true positives, and the specificity, representing the percentage of true negatives [21].

A high sensitivity of distraction detection shows that the method is good in detecting a distraction when there actually is a distraction, while a high specificity shows that the method does not detect a distraction when there is no distraction. A low sensitivity hints at too many missed distractions, while a low specificity indicates too many falsely detected distractions where no distraction was present in reality.

$$Acc=\frac{true\;pos+true\;neg}{true\;pos+true\;neg+false\;pos+false\;neg}*100$$

$$Sens=\frac{true\;pos}{true\;pos+false\;neg}*100$$

$$Spec=\frac{true\;neg}{true\;neg+false\;pos}*100$$

For the baseline threshold, we used the maximum positive change in TDGV during the first driving segment without secondary task.

### 2.2. Results

#### 2.2.1. Differences in Standard Deviation of Speedometer Gaze Time by Condition

**Figure 5:**
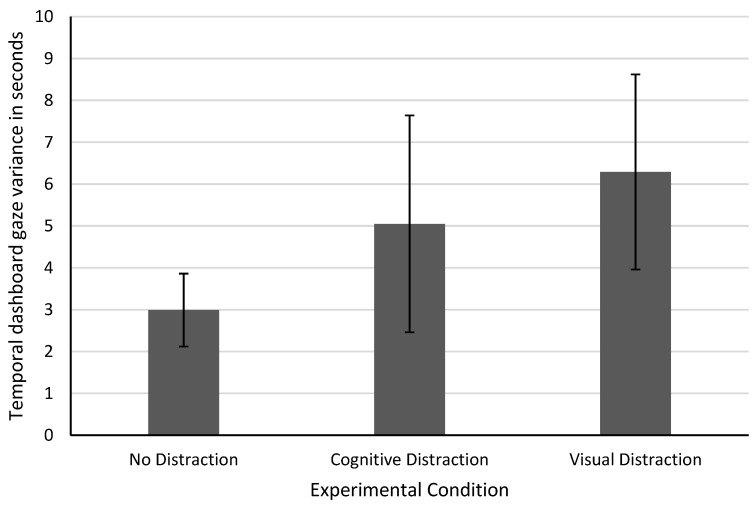
Average temporal dashboard gaze variance over all participants sorted by experimental segments. The error bars represent the standard deviation across different participants.

The first analysis was conducted by calculating the standard deviation of dashboard gaze time for every participant for each whole drive in order to measure global differences in TDGV. To conduct this calculation, the data were aggregated for all participants and grouped by the experimental conditions before calculating the averages of those condition groups. The resulting data in Figure 5 show that cognitively and visually distracted experimental segments show a higher standard deviation than the undistracted driving segments, which indicates that under distraction, the gaze to the speedometer loses its regularity.

The higher TDGV in visual distraction is a result of changed speedometer gaze intervals due to the additional behavioral requirement of looking at the side task. This result is a first support for the hypothesis of this paper. However, visual distraction shows a similar increase in TDGV. This shows that TDGV is not able to differentiate well between cognitive and visual distraction events. While it would be beneficial to differentiate between both distraction types, for the purpose of developing a general driver distraction detection method, it is not necessary. The general focus of this work lies on the development of a system that is generally able to detect cognitive distraction. The differentiation from other distraction types will be considered in future iterations.

#### 2.2.2. Performance of Distraction Detection

**Figure 6:**
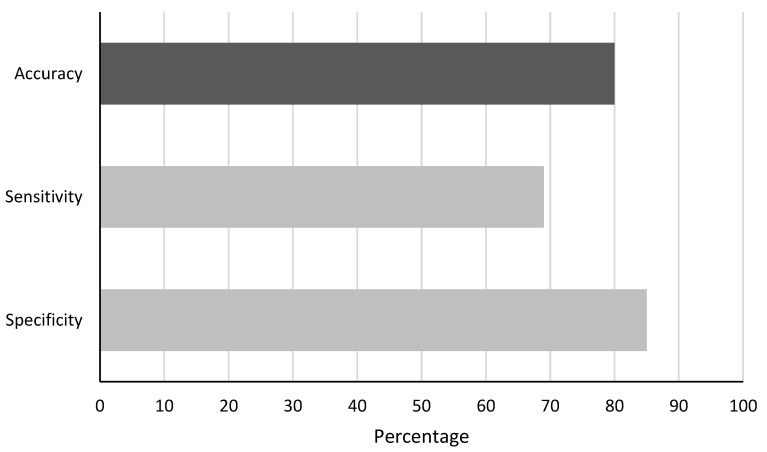
Accuracy, sensitivity, and specificity of distraction detection for cognitive distraction detection side-to-side in study 1.

With the above-described detection method and the explained validation method, the detection method achieved an accuracy of 80%. A comparison of the performance parameters is shown in Figure 6.

This performance is on par with current state-of-the-art machine learning methods, such as from Yang et al. 2020 [22], who developed three models for detecting cognitive distraction with accuracies around 80%. Similarly, the AIDE EU project states that for the purpose of informing other in-vehicle systems, a distraction detection accuracy of 70% is sufficient and 85% is good. However, for direct warning systems, a specificity of 95% is required for the driver to not become frustrated with the system [23]. Therefore, we use those performance parameters as our thresholds.

While comparable to other state-of-the-art models and falling into the AIDE category for “informing other in-vehicle systems”, the accuracy of the cognitive distraction detection method presented here does not yet fulfill the requirement for being a direct warning system. One possible way to approach increasing the accuracy of the system is to investigate the required baseline measurement, as the performance is dependent on the validity of the data measured during this period. The TDGV method assumes a regularity of gaze behavior during the baseline to detect cognitive distraction. It is possible that a varying quality of the baseline due to being distracted during the baseline drive, as well as individual differences in dashboard gaze regularity, could be performance limiting factors. Both would manifest in increased baseline values.

This led us to perform a second study to investigate the quality of the baseline as a limiting factor for TDGV change performance. To increase statistical power to compare different baseline qualities, we increased the sample size of the second study compared to the first. The larger sample size was enabled by conducting the second study in a driving simulator, which also allowed us to control the driving environment more precisely than a field study. The research question for the following study is how much the quality of the baseline influences the performance of the TDGV change distraction detection method.

## 3. Study 2: Investigation How Baseline Influences Accuracy

### 3.1. Materials and Methods

#### 3.1.1. Design

To evaluate the effect the baseline quality and individual differences in dashboard gaze regularity have on the performance of the distraction detection method presented here, we conducted a second study with 41 participants. We chose a larger sample size this time to include more variance in our sample for better detection of the baseline effects. To better obtain more participants, the study was conducted in a driving simulator. Using a driving simulator allowed us to control the environment better and to test more participants.

Participants performed the same cognitive task as in study 1. However, we left the visual distraction task out this time due to the reasons discussed above in Section 2.2.1, namely that the requirement of the iteration of the distraction detection method investigated here is to detect cognitive distraction in general, not to differentiate it from other distraction types. This requirement will be investigated in later iterations.

#### 3.1.2. Study Procedure

**Figure 7:**
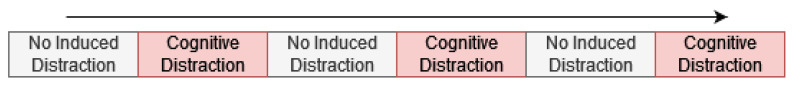
Experimental conditions during study 2. Each segment took approximately one minute.

After being instructed on the study and providing written informed consent for their participation, participants started driving without performing a secondary task; they alternated between driving focused and performing the ACPT (see study 1 materials) while driving. In total, each participant drove for six minutes, three times with the secondary task and three times without. Figure 7 shows a schematic of the experimental segments.

To control for the effectiveness of the distraction task intervention, after the drive, the participants answered how much they were able to focus on the driving task, with and without the secondary task, on a scale between 1 (not focused) to 7 (very focused). Later during analysis, these two values were subtracted from each other to obtain an estimate of how distracting the task was for each participant. The scale of the resulting values ranged from 0 (not distracted) to 6 (very distracted).

$$Distraction\;level=Focus_{without\;task}-Focus_{with\;task}$$

### 3.2. Results

#### 3.2.1. General Performance of Distraction Detection

**Figure 8:**
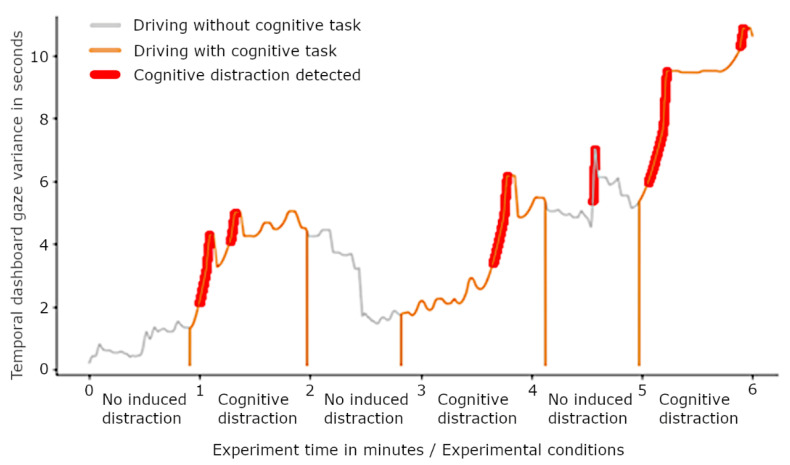
Timeseries graph of detection approach with distraction detection for one example participant. Red, bold marking shows where the metric detected a distraction. The slight variation in the length of the segments results from variations in setting the segment-defining markers during the experiment.

A real data example of how the distraction detection works in one driver is depicted in Figure 8. The graphic shows the TDGV value of a driver during a six-minute trip. The data are segmented in different experimental conditions (no distraction/cognitive distraction). The bold marking indicates when the distraction detection method triggered and detected a cognitive distraction. In this example, the detection method recognized all three cognitive distraction segments and indicated one not-distracted segment as distracted. Possible reasons for such false positives are elaborated on below in the discussion of this paper.

We excluded five participants due to invalid eye tracking data caused by incorrect pupil detection or data logging errors. We then performed a plausibility check for investigating if the cognitive distraction induction worked and to check if our categorizations of cognitive distraction segments are valid. To achieve this, we median split the participants based on their reported distraction level (min = 0, max = 4, median = 2) during the cognitive task. This revealed that the intervention had a distracting effect on the majority of participants except for eight, who were excluded from future analysis. Therefore, our valid sample consisted of 28 participants.

This valid sample showed an overall cognitive distraction detection accuracy of 68%, with a sensitivity of 65% and a specificity of 70%, which is significantly lower than the detection performance in study one. To investigate this issue of performance reduction, we looked into the baseline as a possible cause.

#### 3.2.2. Baseline Quality as a Performance Influencing Cause

To investigate the effect the baseline has on the performance of the method, we first defined the quality of the baseline such that a good baseline shows little TDGV change, since this would indicate either distraction during the baseline or that a driver generally shows very little regularity in their gaze behavior.

To formalize this quality definition, the maximum TDGV change during the baseline was z-transformed by the whole TDGV change data of a participant during the whole drive. This resulted in a value representing how many standard deviations the maximum TDGV change during the baseline differs from the average TDGV during the whole drive.

With this z-transformed value, participants were again median split (min = −1.05, max = 11.9, median = 0.65), where the group with the higher value represented the lower baseline quality and vice versa.

**Figure 9:**
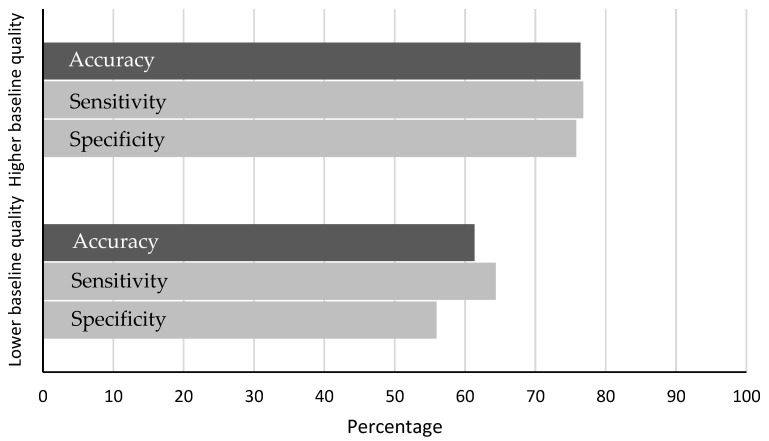
Differences between groups with a lower and higher baseline quality in distraction detection performance parameters.

Figure 9 shows the difference between both baseline quality groups on the performance of the distraction detection. Drivers with a higher quality baseline show an accuracy of 76%, with 77% sensitivity and 76% specificity, which is closer to the values measured in the first study.

## 4. Discussion

### 4.1. Accuracy of Temporal Dashboard Gaze Variance (TDGV) Change Metric

The accuracy of detecting segments of cognitive distraction with the TDGV change distraction detection method, when not including participants based on a low pre-measured baseline quality, lies between 76% and 81% correct detection. When including those participants, the accuracy lies at 68%. This decrease of 8–13% indicates the importance of a well-conducted baseline for the performance of the method and poses a limitation of the method. We came up with two reasons that might facilitate a reduced baseline quality.

(a) Because of its similarity to external cognitive distraction, we assume that TDGV change also detects mind-wandering in low cognitive load, which we did not account for in our studies. This means that some drivers could have been distracted during the baseline drive due to mind-wandering.
(b) The second reason could be individual differences in drivers’ general gaze regularity causing a general lack of regularity in some drivers.

While reduced baseline quality due to mind-wandering can potentially be accounted for by making sure the driver focuses on driving during the baseline, a reduction due to individual gaze irregularities means that the detection method works better for people who generally show more regular gaze behavior than for people with very irregular gaze patterns. Future iterations should find ways to account for that, possibly by incorporating general gaze regularity during the baseline as a moderator.

### 4.2. Comparison to Current Research

The accuracy of the currently presented metric is in line with requirements proposed by the AIDE EU project, which states that 70% accuracy is sufficient and 85% is good to effectively inform an in-vehicle decision logic about a driver’s distraction state [23]. For drivers showing regular gaze patterns, the performance is comparable to other state-of-the-art cognitive distraction detection methods, which achieve accuracies ranging from 70% to 83.5% [2,14,20]. For drivers with generally irregular gaze patterns, the algorithm’s performance is slightly below those other methods, again emphasizing the need to find a way to control for such behavioral patterns.

Considering the cognitive distraction detection method presented here works only with one parameter and consists of predefined rules rather than a machine learning inference, the accuracy is promising for future iterations and systems implementing this method.

### 4.3. Limitations of Studies and Suggested Next Steps

While study 1 was an exploratory field study without much control, we designed for controlled experimental conditions in study 2, and we assessed general distraction levels during the experiment. However, there cannot be full certainty that participants’ focus was aligned with the experimental segments. As already stated, we assume that TDGV change also detects mind-wandering in low cognitive load, which we did not account for in our studies. This means, that participants could have been distracted by internal mind-wandering effects during segments without a secondary task, which the method probably detected. Our experimental design, however, would count those detections as false positives, even if they are correctly detected distraction events.

This assumption is supported by the difference in specificity between study 1 (85%) and 2 (69–75%) of 10–16%. The driving task in the field study (study 1) was more complex, required more attention, and was probably taken more seriously than in the simulator study (study 2), leading to constant higher driving-relevant cognitive load and, therefore, less room for mind-wandering.

A solution for this would be the collection of cognitive distraction data in the form of electroencephalographic (EEG) methods to use as a ground truth base to check the TDGV data against. Such systems are able to directly infer and differentiate mind-wandering, external cognitive distraction, and the level of driving task attendance in the driver [24,25]. EEG systems can only be used in experimental settings due to their cumbersome deployment, which heavily restricts their use in real detection systems. However, they are ideal for experimental scientific purposes to validate methods, such as the TDGV change distraction detection.

## 5. Conclusions

The best-case accuracy of the eye tracking based cognitive distraction detection method presented here lies between 76% and 81%, while its average accuracy is around 69%. Comparing this performance with the threshold for a good driver monitoring system of 70–85%, as proposed by the AIDE EU project [23], indicates that the change in temporal dashboard gaze variance (TDGV) is a promising measurement metric for cognitive distraction if used thoughtfully and if its limitations are considered. The performance is especially promising considering that the model presented here is relatively simple compared to other methods, as it only uses a single parameter for the whole detection process and does rely on a set of hand-crafted rules.

However, the performance of the detection method is strongly dependent on the quality of the individual driver’s baseline measurement and individual differences in gaze regularity. While the algorithm works satisfyingly as a first iteration in participants with rather regular gaze patterns, it does not achieve the 70% threshold for a satisfying detection system in participants with very irregular gaze behaviors or a low-quality baseline measurement. To counteract this limitation and to increase performance further, future iterations will utilize data-driven machine learning algorithms informed by the present results and will incorporate additional gaze parameters derived from other detection approaches. Additionally, future studies should include ground truth data about cognitive distraction and mind-wandering to more precisely assess the performance of the distraction detection.

## Data Availability

All code and data used to generate the results in the study can be requested from Cyril Marx (cyril.marx@v2c2.at) when providing an appropriate reason.
